# Autoantibodies against PIP4K2B and AKT3 Are Associated with Skin and Lung Fibrosis in Patients with Systemic Sclerosis

**DOI:** 10.3390/ijms24065629

**Published:** 2023-03-15

**Authors:** Marija Geroldinger-Simić, Shaghayegh Bayati, Emmie Pohjanen, Norbert Sepp, Peter Nilsson, Elisa Pin

**Affiliations:** 1 Department of Dermatology and Venereology, Ordensklinikum Linz Elisabethinen, 4020 Linz, Austria; 2 Faculty of Medicine, Johannes Kepler University, 4040 Linz, Austria; 3 Department of Protein Science, KTH Royal Institute of Technology, SciLifeLab, 171 65 Stockholm, Sweden

**Keywords:** systemic sclerosis, skin fibrosis, lung fibrosis, biomarkers, autoantibody profiling, protein array

## Abstract

Systemic sclerosis (SSc) is a rare autoimmune systemic disease that leads to decreased survival and quality of life due to fibrosis, inflammation, and vascular damage in the skin and/or vital organs. Early diagnosis is crucial for clinical benefit in SSc patients. Our study aimed to identify autoantibodies in the plasma of SSc patients that are associated with fibrosis in SSc. Initially, we performed a proteome-wide screening on sample pools from SSc patients by untargeted autoantibody screening on a planar antigen array (including 42,000 antigens representing 18,000 unique proteins). The selection was complemented with proteins reported in the literature in the context of SSc. A targeted antigen bead array was then generated with protein fragments representing the selected proteins and used to screen 55 SSc plasma samples and 52 matched controls. We found eleven autoantibodies with a higher prevalence in SSc patients than in controls, eight of which bound to proteins associated with fibrosis. Combining these autoantibodies in a panel could lead to the subgrouping of SSc patients with fibrosis. Anti-Phosphatidylinositol-5-phosphate 4-kinase type 2 beta (PIP4K2B)- and anti-AKT Serine/Threonine Kinase 3 (AKT3)-antibodies should be further explored to confirm their association with skin and lung fibrosis in SSc patients.

## 1. Introduction

Systemic sclerosis (SSc) is a chronic systemic disease that leads to decreased survival and quality of life due to skin and/or internal organ fibrosis, vasculopathy, and autoimmune inflammation. SSc is a rare disease that manifests worldwide, mainly in adult women, but men and children can also be affected [1]. Patients with SSc can develop digital ulcers, reduced mobility due to skin sclerosis, dysphagia, reflux due to fibrosis of the esophagus, or dyspnea due to lung and heart involvement. The primary causes of death in SSc are lung fibrosis and pulmonary arterial hypertension (PAH) [2]. An early diagnosis is crucial for the clinical benefit of SSc patients.

Autoantibodies, such as anti-Scl70 and anti-centromeric antibodies play a significant role in the diagnosis of SSc [3]. However, 5–10% of SSc patients remain negative to these markers [4,5]. Moreover, autoantibodies have been associated with clinical manifestations. For instance, anti-RNA polymerase III-antibody has also been considered as specific for SSc and associated with the risk of renal crisis [6]. Several autoantibodies have been associated with lung fibrosis [7,8,9,10], but none is currently used in the clinical setting. Therefore, there is an urgent need for disease-selective autoantibodies that could serve as biomarkers to improve the diagnosis and subclassification of SSc.

Our primary aim in this study was to perform a broad autoantibody profile to identify autoantibodies in plasma of SSc patients that are associated with skin and lung fibrosis and might serve as potential diagnostic biomarkers of SSc or help in stratifying SSc patients in the future. To achieve this, we applied an in-house developed protein array technology based on the Human Protein Atlas collection of human protein fragments [11]. The well-established technology has been successfully applied to profile the autoantibody repertoire within several diseases [12,13,14], including autoimmune inflammatory conditions [15,16], as well as in healthy individuals [17].

This study represents a nearly proteome-wide autoantibody screening performed in plasma of patients with SSc and matched controls.

## 2. Results

### 2.1. Increased Autoantibody Load in SSc Patients with Skin and Lung Fibrosis

**Figure 1:**
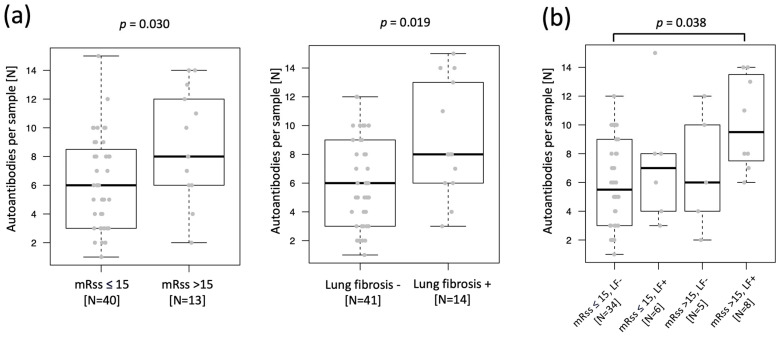
Autoantibody load in SSc patients with lung and skin fibrosis. The box plots show the increase in the number of autoantibodies per sample (autoantibody load) in SSc patients with (**a**) mRSS > 15 or lung fibrosis, and particularly in (**b**) SSc patients presenting both mRSS > 15 and lung fibrosis. Herein, *p*-values refer to the (**a**) Mann–Whitney–Wilcoxon and (**b**) Kruskal–Wallis tests; *p* < 0.05, cutoff for significance; mRSS: Modified Rodnan skin score; LF: Lung fibrosis.

The initial untargeted screening of two plasma sample pools, one including four SSc patients with diffuse SSc (dcSSC) and the other one including four SSc patients with localized SSc (lcSSC) (Supplementary Table S1) resulted in the selection of 59 proteins (unique protein IDs) with higher IgG reactivity in the dcSSc pool compared to the lcSSc pool. These proteins were included in the targeted screening, where the whole study sample set (55 patients with SSc and 52 controls; Table 1) was tested using a bead array including 246 antigens. The 246 antigens included 73 antigens representing the 59 unique proteins selected by untargeted screening on planar array, plus 173 antigens representing 136 unique proteins selected from literature (Supplementary Figure S1 and Table S2). When available, the targeted screening included more than one antigen (i.e., protein fragment) per protein to cover the greatest extent possible of the protein sequence. IgG antibodies were detected toward 132 out of 246 tested antigens (54%), with each antigen-specific antibody detected in one to forty-two samples. Single samples were positive to one to fifteen autoantibodies. We evidenced a higher, even though not statistically significant (*p* = 0.06), median number of autoantibodies per sample (autoantibody load) in SSc patients compared to controls (Supplementary Figure S2). We tested whether this difference may be affected by the difference in age between the two groups (Table 1), and the analysis showed no correlation between autoantibody load and age in SSc patients (r = 0.04, *p* = 0.76) or controls (r = 0.21, *p* = 0.13). When comparing subgroups of SSc patients, we identified an increase in autoantibody load in patients with active skin (mRSS above 15) and lung fibrosis, with the highest autoantibody load detected in patients presenting both features (Figure 1). None of the other clinical characteristics (calcinosis cutis, digital ulcers, dysphagia, Raynaud, reflux, and sicca) could be linked to any autoantibody load increase. Positivity for clinically tested autoantibodies (anti-Scl70, anti-centromere, anti-SSA, anti-Rnp/Sm) was also not linked to any general increase in autoantibody load in our array analysis.

### 2.2. Selection of 11 Autoantibodies with Higher Prevalence in Plasma of SSc Patients and Reactive toward Fibrosis-Related Proteins

**Figure 2:**
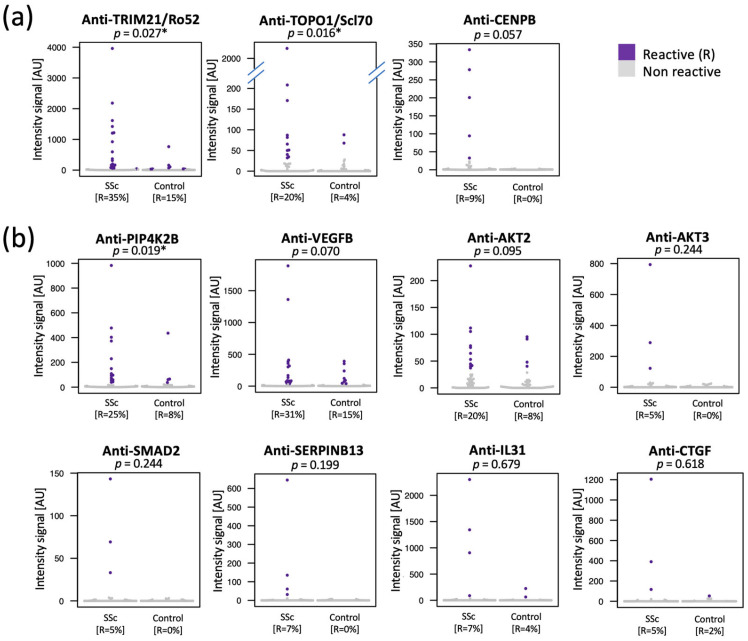
Autoantibodies with higher prevalence in SSc patients. The panel collects the 11 autoantibodies showing higher prevalence in plasma of SSc patients compared to controls. (**a**) Three of the autoantibodies are already well-known in the context of SSc. (**b**) Eight autoantibodies bound to proteins involved in fibrosis. The dot plots show the intensity signals detected for each autoantibody in each sample. Each dot represents one sample. Purple dots indicate samples where the specific autoantibody passed the cutoff for reactivity, and therefore, are defined as reactive. The *p*-values refer to Fisher´s Exact test; [*] is used for *p* < 0.05, cutoff for significance.

We performed a case versus control analysis for prevalence (Fisher´s Exact test) and selected autoantibodies for further investigation in our study (Figure 2). The criteria of selection were for the autoantibodies to show a higher statistically significant prevalence in SSc patients compared to controls (*p* < 0.05), or a higher even though not statistically significant prevalence in SSc but binding to a protein known to be involved in fibrosis. Eleven autoantibodies passed these criteria for selection. Three of these bound to well-known autoantibody targets in SSc (Figure 2a): Anti-DNA topoisomerase 1 antibodies (anti-TOPO-1/Scl70; *p* = 0.016), anti-Tripartite motif containing 21 antibodies (anti-TRIM21/Ro52; *p* = 0.027), and Centromere Protein B (CENPB; *p* = 0.057), all showing a higher statistically significant prevalence in SSc compared to the controls. The remaining eight autoantibodies bound to proteins involved in fibrotic processes (Figure 2b): Phosphatidylinositol-5-phosphate 4-kinase type 2 beta (PIP4K2B; *p* = 0.019), Vascular endothelial growth factor B (VEGFB), AKT Serine/Threonine Kinase 2 (AKT2), AKT Serine/Threonine Kinase 3 (AKT3), SMAD Family Member 2 (SMAD2), Serpin Family B Member 13 (SERPINB13), Interleukin 31 (IL31), and Connective Tissue Growth Factor (CTGF). Five of these proteins are part of (AKT2, AKT3, SMAD2) or target genes (CTGF, IL31) of the TGF-beta pathway.

### 2.3. High Prevalence of Anti-PIP4K2B, Anti-AKT3, Anti-TRIM21/Ro52, and Anti-CENPB in Subgroups of SSc Patients

We evaluated the prevalence of the 11 selected autoantibodies (Figure 2) in subgroups of SSc patients with specific clinical features, as well as in SSc patients negative for known autoantibody markers, respectively to find potential associations, especially with skin and lung fibrosis and to identify new autoantibodies that could improve the diagnosis of autoantibody negative SSc patients in the future.

**Figure 3:**
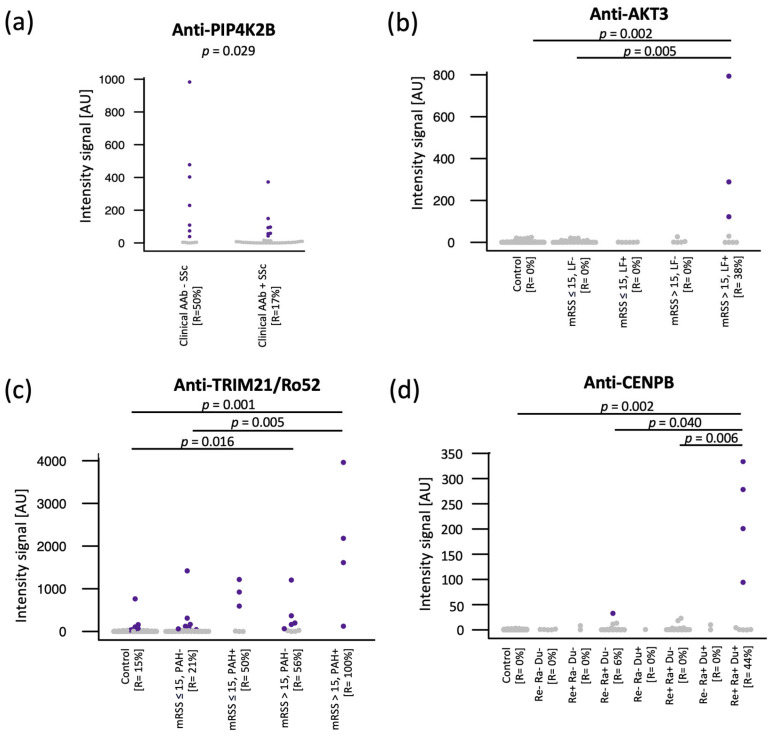
Autoantibodies associated with subgroups of SSc patients. (**a**) Anti-PIP4K2B antibodies were detected in 7/14 (50%) of the SSc patients negative for the clinical autoantibody (AAb) panel: anti-TOPO-1/Scl70, anti-centromere, anti-Rnp/Sm, anti-Rnp70, anti-SSA/Ro, anti-SSB/La, and anti-Sm in clinical tests. (**b**) Anti-AKT3 autoantibodies were detected at high levels in SSc patients with lung fibrosis (LF) and active skin fibrosis with mRSS > 15. (**c**) Anti-TRIM21/Ro52 showed the highest prevalence (100%) and intensity in SSc patients with both PAH and mRSS > 15. (**d**) Anti-CENPB antibodies were detected at high levels in patients with digital ulcers (Du), Raynaud (Ra), and reflux (Re). Each dot represents one sample. Purple dots indicate samples where the autoantibody passed the cutoff of reactivity. Group comparisons were carried out by Fisher´s Exact test. *p* < 0.05, cutoff for significance; mRSS: Modified Rodnan skin score.

Anti-PIP4K2B antibodies have been detected in 50% (seven out of fourteen) of the SSc patients negative to the autoantibody panel routinely measured at our clinic, including anti-TOPO-1/Scl70 and anti-centromere (Figure 3a). Adding anti-PIP4K2B to the clinical autoantibody panel increased the SSc positivity of our cohort from 75% to 87%.

Anti-AKT3 showed higher prevalence in patients with lung fibrosis (*p* = 0.014) and in patients with active skin fibrosis (i.e., mRSS score > 15, *p* = 0.012). We, therefore, combined SSc patients into four categories: without lung fibrosis and with mRSS score below or equal to 15 (LF+ and mRSS ≤ 15), without lung fibrosis and with mRSS score above 15 (LF+ and mRSS > 15), with lung fibrosis and mRSS below or equal to 15 (LF+ and mRSS ≤ 15), and with lung fibrosis and mRSS score above 15 (LF+ and mRSS > 15). Anti-AKT3 antibodies have been detected in 38% (three out of eight) of patients with both lung fibrosis and mRSS score > 15, but in no other subgroup (Figure 3b).

Anti-TRIM21/Ro52 antibodies showed higher prevalence in SSc patients with mRSS score > 15 (*p* = 0.007), and in patients with PAH (*p* = 0.023). When combining these two clinical features, patients presenting both mRSS score > 15 and PAH showed the highest prevalence of anti-TRIM21/Ro52 antibodies (100%) (Figure 3c).

Finally, anti-CENPB antibodies were shown to be present in 44% (four out of nine) of SSc cases with reflux, Raynaud, and digital ulcers, while almost completely absent in SSc subgroups with only one or two of these clinical manifestations (Figure 3d).

### 2.4. Autoantibody Combination and Cluster Analysis Separate Subgroups of SSc Patients with Skin and Lung Fibrosis

Previously, we identified single autoantibodies with higher prevalence in specific clinical subgroups. However, the combination of several autoantibodies could improve the subgroups separation. With this aim, we tested the performance of the combination of 11 autoantibodies to discriminate between SSc patients and controls using cutoffs of one to three autoantibodies per sample. We applied a ROC curve analysis, which resulted in an area under the curve (AUC) of 0.8, and therefore, demonstrated a good performance of the 11 autoantibodies in separating the two groups (Supplementary Figure S3). The curve represents the true positive and false positive rates at each of the applied cutoffs, for which we also include detailed numbers in Supplementary Table S3. Setting a cutoff of at least two autoantibodies per sample, 53% of the SSc patients were classified as positive and 90% of the controls as negative, while with a cutoff of at least three autoantibodies per sample, 25% of the SSc cases were classified as positive and 100% of the controls as negative.

**Figure 4:**
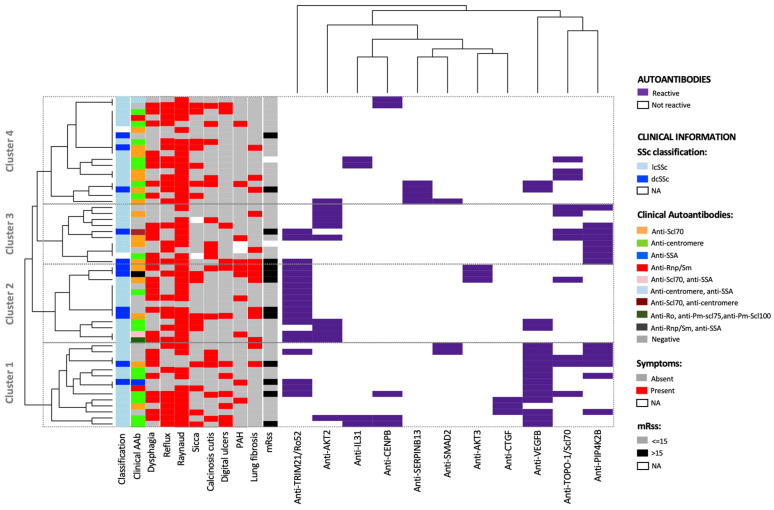
Heatmap and cluster analysis of the 11 selected autoantibodies in plasma of SSc patients. The cluster analysis revealed four main clusters corresponding to patient subgroups with different autoantibody signatures. Cluster 1 included most samples with anti-VEGFB antibodies, many of which have calcinosis and digital ulcers. Cluster 2 contained most samples with anti-TRIM21/Ro52, many of which have lung fibrosis and PAH. Cluster 3 included half of the SSc patients with anti-PIP4K2B, while cluster 4 had the lowest prevalence of autoantibodies among the identified clusters. Each row in the heatmap represents one SSc individual. Purple rectangles indicate the autoantibodies detected in each sample.

A cluster analysis of the autoantibody reactivities did not evidence any separation of patients with localized and diffuse SSc. On the other hand, we identified four clusters evidencing some degree of separation of the patients based on specific clinical features (Figure 4). The majority (70%, 9/13) of the patients with mRSS score > 15 was included in clusters 1 or 2, mainly driven by anti-VEGFB and anti-TRIM21/Ro52 antibodies, respectively. Moreover, the comparison between clusters 1 and 2 evidenced that cluster 1 included a higher number of patients with skin involvement—digital ulcers (43% vs. 15%) and calcinosis cutis (36% vs. 15%)—while cluster 2 included more patients with lung involvement—lung fibrosis (46% vs. 14%) and PAH (31% vs. 15%). In accordance with the higher prevalence of patients with digital ulcers, cluster 1 also showed a higher prevalence of patients with anti-centromere antibodies than cluster 2 (43% vs. 23%). Three of the patients positive to anti-centromeric antibodies and with digital ulcers in cluster 1, were shown to be positive for anti-CENPB, one of the main centromeric targets of autoantibodies, using our antigen bead-array technology.

In cluster 1, anti-VEGFB mainly overlapped with anti-PIP4K2B, which were absent in cluster 2. In cluster 2, rather, anti-TRIM21/Ro52 mainly overlapped with anti-AKT2 and anti-AKT3 antibodies.

Cluster 3 was mainly driven by anti-PIP4K2B and anti-AKT2 antibodies. No specific clinical feature could be linked to this pattern, except that none of the patients in this group was affected by sicca. Cluster 4 included the SSc patients with the lowest degree of positivity to the 11 autoantibodies.

## 3. Discussion

In this study, we aimed to identify autoantibodies that may be associated with skin or lung fibrosis and/or that might improve the diagnosis and/or subclassification of SSc in future. The major results of our high-multiplexing autoantibody screening of 55 plasma samples from SSc patients and 52 controls by means of antigen arrays showed 11 autoantibodies with increased prevalence in SSc patients compared to controls, eight of which bound to fibrosis-associated proteins: PIP4K2B, VEGFB, AKT2, AKT3, SMAD2, SERPINB13, IL31, and CTGF. Our data indicated anti-PIP4K2B and anti-AKT3 as the most promising candidates for further investigation as potential fibrosis-associated autoantibodies in SSc patients. We also evidenced that combining autoantibodies in panels may help in improving the subclassification of SSc patients with skin and lung fibrosis in future.

The case versus control analysis evidenced a trend with increased autoantibody load (median number of antibodies per sample) in SSc patients compared to controls (Supplementary Figure S2). This difference was not influenced by the difference in age of two groups, in accordance with previously published data [18]. Furthermore, our data showed that the presence of lung fibrosis and active skin fibrosis in SSc is characterized by an increase in autoantibody load (Figure 1). Previously published data showed that increased autoantibody load in sputum in the early stages of the rheumatoid arthritis patients may be associated with lung involvement [19]. Moreover, changes in autoantibody load in systemic lupus erythematosus (SLE) during the natural course of the disease or due to effects of the therapy have been described [20]. Therefore, further studies are needed to explore the significance of the autoantibody load for the diagnosis of SSc or the presence of lung involvement in SSc.

Among the 11 selected autoantibodies, anti-TOPO-1, anti-TRIM21, and anti-CENPB are already known and routinely used in the context of SSc. We could also detect eight previously unpublished autoantibodies in the context of SSc. These autoantibodies bound to the fibrosis-related proteins PIP4K2B, VEGFB, AKT2, AKT3, SMAD2, SERPINB13, IL31, and CTGF. Therefore, fibrosis during SSc might not only induce a general increase in the autoantibody load of patients, but also lead to the generation of autoantibodies directed toward proteins that participate in fibrosis.

We identified anti-PIP4K2B to be significantly more prevalent in SSc patients compared to controls (Figure 2b). Our data further showed that anti-PIP4K2B antibodies were detected in half of the SSc cases classified as negative to anti-TOPO-1/Scl70 and anti-centromere (Figure 3a). Adding anti-PIP4K2B antibody to autoantibody markers routinely used at our clinic, increased the SSc positivity from 75% to 87%. Therefore, antibodies to PIP4K2B may contribute to the diagnosis of SSc, and this should be further evaluated in multi-center studies in future. The role of PIP4K2B protein and anti-PIP4K2B antibodies in the pathophysiology of SSc is not known. The PIP4K2B enzyme regulates lipid metabolism in fibroblasts and a decreased PIP4K2B expression leads to fibrosis due to the enhancement of TGF-β [21,22,23,24]. Further studies are needed to explore the role of anti-PIP4K2B antibodies in fibrosis during SSc.

We found autoantibodies toward VEGFB with a higher prevalence in patients with SSc compared to controls (Figure 2b). Even though the difference was not statistically significant, the prevalence in SSc was twice as high as in controls, and the autoantibody target was shown to be interesting on a biological point of view. VEGFB is relevant for the function of newly built vessels [25] and is involved in endothelial transport of fatty acids and lipid accumulation in muscles or kidney [25,26,27]. Furthermore, VEGF is increased during hypoxia and is involved in the development of lung fibrosis and pulmonary arterial hypertension [28,29,30]. VEGF inhibitor, nintedanib, was recently approved for the therapy of lung fibrosis in patients with SSc [31]. Therefore, further studies are needed to explore the possible role of VEGFB-specific antibodies in fibrosis during SSc.

Our study identified a higher, not statistically significant, prevalence of antibodies against AKT2 and AKT3 in SSc patients as compared to controls (Figure 2b). The prevalence of anti-AKT3 antibodies was significantly increased in SSc patients with lung fibrosis and mRSS score > 15 (Figure 3b). AKTs are involved in the pathophysiology of SSc by stimulating fibrosis as part of TGF-ß signaling [32,33]. Furthermore, AKT2 and AKT3, as part of phosphoinositide 3-kinase (PI3K)/AKT/mTOR signaling pathway, have been associated with several adenocarcinomas [34]. As the risk for breast and lung carcinoma is increased in patients with SSc [35,36,37,38], we could hypothesize that anti-AKT antibodies might be relevant to define a subgroup of SSc patients with increased risk for cancer. Further studies are needed to explore the role of anti-AKT antibodies as biomarker candidates for fibrosis and risk of cancer in SSc patients.

Our study also found autoantibodies targeting SMAD2, SERPINB13, CTGF, and IL31 in small subsets of SSc patients (Figure 2b). Even though the numbers are small, we hypothesized that these data were still worth further consideration as, interestingly, all four targets are involved in fibrosis; SMAD2 as part of the TGF-ß pathway along with AKT2 and 3, and CTGF and IL31 as target genes of the TGF-β pathway [39,40]. Anti-CTGF antibodies showed antifibrotic effects in mice models of systemic sclerosis [41]. Anti-serpin antibodies reduced autoimmune inflammation in diabetic mice [42]. IL31 is a Th-2-associated cytokine and was previously described as a candidate biomarker of skin and lung fibrosis in a subset of SSc patients [43]. Moreover, blockade of IL31 due to IL31-receptor antibody led to the amelioration of fibrosis in a mouse model of SSc [43,44]. Further studies are needed to explore the role of these autoantibodies in the fibrosis and pathogenesis of SSc.

In addition, IL31 is known as the mediator of pruritus and has been shown to be elevated in atopic dermatitis and psoriasis [45]. Recently, nemolizumab, a humanized monoclonal antibody targeting the IL31 receptor, has been proven efficient in clinical studies for prurigo nodularis [46]. As an itch can be very intensive and often decreases the quality of life in patients with SSc, further studies should explore the role of IL31 and anti-IL31 antibodies for pruritus in SSc patients.

We found that combining the 11 identified autoantibodies allowed us to correctly classify 25–53% of the SSc patients and 90–100% of the controls (Supplementary Table S2 and Figure S3). Furthermore, the cluster analysis evidenced how certain antibodies seem to preferably associate with a cluster of patients with certain clinical features (Figure 4). Among the clusters, cluster 1, mainly driven by anti-VEGFB antibodies, showed the highest frequency of SSc patients presenting digital ulcers. Literature data report that impaired angiogenesis markers, including VEGF, have a predictive value for the occurrence of digital ulcers [47]. On the other hand, cluster 2 is mostly driven by anti-TRIM21/Ro52 antibodies and showed the highest frequency of patients with lung fibrosis and PAH. Anti-TRIM21/Ro52 was previously reported to be associated with interstitial lung disease [48]. Furthermore, the combination of 11 autoantibodies could not separate patients with limited SSc from patients with diffuse SSc. This is in line with previous results, showing that clustering of SSc patients based on skin changes may not depict the complete spectrum of the SSc; therefore, involvement of other organs, as well as different autoantibodies, should be taken into consideration to define subgroups of SSc patients [49,50]. Based on the cluster analysis, we believe anti-VEGFB antibodies and anti-TRIM21/Ro52 antibodies should be further investigated in the context of digital ulcers and lung fibrosis and/or PAH in SSc patients.

We also need to consider that our study presents limitations. First, we analyzed the plasma of SSc patients in a single small cohort and at a single time point. Therefore, our data need to be validated in independently collected and larger sample cohorts. Moreover, longitudinal analyses in larger multi-center cohorts and evaluation of results associated with the medication should be included in future studies. Future verification studies should also complement the current data by including a broader selection of non-healthy controls, including patients with inflammatory and fibrotic autoimmune diseases other than SSc, such as patients with interstitial lung disease (ILD) other than SSc-ILD. Data on the extent of SSc-ILD are also needed to complement the current data. On a target level, while our study included the major autoantibodies used for diagnosis of SSc, future studies should also complement these data with the evaluation of positivity for other autoantibodies reported to be associated with SSc (such as anti-U3RNP, anti-fibrillarin, anti-Th/To, anti-PmScl, anti-RNA polymerase III, anti-Ku, anti-PmScl, and anti-U1RNP antibodies). On a technological level, our large collection of protein fragments allowed us to include in the study representations of all human proteins. However, by definition, protein fragments represent only a portion of the protein´s sequence, and therefore, some potentially reactive epitopes might have been missed in the current analysis; therefore, it would be interesting to include in future verification studies additional protein fragments from each of the selected proteins and/or full-length proteins to increase the protein representativeness. Moreover, the target selection performed by planar antigen array has been limited by being based on two pools of plasma samples each representing only four patients with dcSSc or lcSSc. Although this reduces the representativeness of the pool for the whole patient group, it also enables us to avoid an excessive sample dilution that might cause the loss of signals from autoantibodies present at low prevalence or concentration. In fact, these autoantibodies may still be useful to identify interesting patient subgroups. Our array includes an anti-human IgG antibody that we use as the control of sample loading and allows us to ensure that we have a good level of human IgG in the tested samples. This analyte usually results in signals at saturation level, and therefore, prevents us from running a correlation between the total IgG and the autoantibody load, which would be interesting to perform.

In summary, this study selected eleven autoantibodies in the plasma of SSc patients, eight of which target proteins linked to fibrosis (PIP4K2B, VEGFB, AKT2, AKT3, SMAD2, SERPINB13, IL31, and CTGF), with some part of the TGF-beta pathway. We do not know whether these autoantibodies are pathogenic or only an epiphenomenon due to the response of the immune system to fibrotic processes in SSc. While this remains to be clarified, this study provides a selection of autoantibodies to be further tested for their association with clinical manifestations of SSc: (i) Autoantibodies against PIP4K2B and AKT3 should be further explored for their association with fibrosis in SSc patients; (ii) anti-PIP4K2B should be further analyzed to confirm its potential to complement anti-Scl70- and/or anti-centromeric antibodies at diagnosis; (iii) autoantibody combinations including anti-PIP4K2B, VEGFB, AKT2, AKT3, and TRIM21/Ro52 should be further investigated for their association with specific clinical symptoms (skin and lung involvement). Finally, an evaluation of a general increase in autoantibodies targeting the TGF-beta pathway should be also further evaluated in broader sample sets.

In conclusion, our study provides new candidate autoantibodies, that should be further evaluated, as single or in combination, for their association with fibrosis and their role for the diagnosis and/or subclassification of SSc in future.

## 4. Materials and Methods

### 4.1. Study Group

The study group included 55 patients with SSc and 52 controls without SSc and was recruited at the Department of Dermatology, Ordensklinikum Linz, Austria. All study participants gave written informed consent. The study has been approved by the Ethics Committee of the Johannes Kepler University Linz, Austria (protocol 1265/2019 and amendments). Table 1 summarizes the clinical characteristics of the study group.

We recruited 46 (84%) female and 9 (16%) male SSc patients vs. 39 (75%) female and 13 (25%) male controls. The median age in SSc patients was 61 (range 25–85) vs. 53 (range 21–79) in the control group (*p* = 0.004). The difference in age of the two groups is due to recruitment limitations during the SARS-CoV-2 pandemic, when the study started.

Of the SSc patients, 42 had limited SSc, 11 had diffuse SSc, and 2 were without skin sclerosis. The mRSS threshold of 15 has been used to subgroup the SSc patients, in line with previously published data [51,52,53]. The clinical routine diagnostic included measurement of a clinical antibody panel with anti-nuclear antibodies (ANA) and ANA-subsets (anti-centromere, anti-Scl70, anti-Rnp/Sm, anti-Rnp70, anti-SSA/Ro, anti-SSB/La, anti-Sm antibodies) refers to standardized ELISA and immunofluorescence assays. Out of 55 SSc patients, 18 were positive for anti-Scl70 antibody, 19 for anti-centromere antibody, and 14 were negative for ANA-subset antibodies.

Lung fibrosis (LF) was evaluated using high-resolution computed tomography (HRCT) scans and pulmonary function tests. Pulmonary arterial hypertension (PAH) was assessed using stress echocardiography and right heart catheterisation.

The control group included 35 patients with an inflammatory disease, such as psoriasis, lupus erythematosus, sarcoidosis, lichen ruber, atopic dermatitis, and 17 patients with a non-inflammatory disease, such as basalioma, lipoma, and venous insufficiency.

Plasma samples for autoantibody profiling using protein arrays were stored at 4 °C for a maximum of 4 h upon collection and then at −80 °C until further processing (the same protocol has been used for all samples included in the study).

### 4.2. Study Design

The presented study aimed to screen autoantibodies from plasma of SSc patients to identify single autoantibodies or signatures that are associated with skin or lung fibrosis and might improve the diagnosis and/or subclassification of SSc in future. The study has been designed in two phases, untargeted and targeted autoantibodies screening (Supplementary Figure S1). Initially, an untargeted autoantibody screening was performed on sample pools from 4 SSc patients with lcSSc and 4 SSc patients with dcSSc using a nearly proteome-wide planar antigen array. Antigens targeted by IgG were selected, and the selection was complemented with proteins reported in the literature in the context of SSc. A targeted antigen bead array was then generated with protein fragments representing the selected proteins and used to test 55 SSc plasma samples and 52 sex-matched controls. The generated data were analyzed to identify single autoantibodies or combinations of autoantibodies (autoantibody panels) with the potential to improve the diagnosis or subclassification of SSc, especially the patients with skin and/or lung fibrosis.

### 4.3. Untargeted Autoantibody Screening by Proteome-Wide Planar Antigen Array

Initially, a nearly proteome-wide autoantibody screening was performed on sample pools from SSc patients with limited disease (lcSSc) and diffuse disease (dcSSc) to select proteins targeted by human IgG antibodies. In detail, two plasma pools from dcSSc and lcSSc, each including four samples from the same number of patients, were tested with our planar antigen arrays, including 42,000 protein fragments representing 18,000 unique proteins [54]. The assay was performed as previously described to identify IgG binding to human protein fragments [14]. Shortly, each pool was diluted 1:100 in assay buffer composed of PBS 0.1% (*v*/*v*) Tween 20 (Thermo Fisher Scientific, Waltham, MA, USA), 3% bovine plasma albumin (Saveen Werner, Limhamn, Sweden), 5% (*v*/*v*) skim-milk powder (Sigma-Aldrich, St. Louis, MO, USA), and 160 µg/mL His6ABP, and incubated for 15 min at room temperature. This step allows for the blocking of any antibodies in the sample that may bind to the His6ABP tag present in all antigens, and that could mask the IgG binding to the protein fragment. The tag is composed of 6 histidine residues and an albumin binding protein of streptococcal origin and is used for affinity purification of the protein fragment at production. The sample (100 µL) was then loaded on the microarray slide and incubated for 1 h at room temperature. After washing the sample excess with PBS-T 0.1%, the array was incubated for 1 h with 1:40,000 hen anti-His6ABP IgY (Immunotech HPA, Stockholm, Sweden) for detection of the microarray spots. After additional washes with PBS-T 0.1%, the arrays were incubated for 1 h with fluorescently labelled detection antibodies: Goat anti-chicken IgY Alexa Fluor^®^ 555 (A21437, Invitrogen, Waltham, MA, USA) and goat anti-human IgG (H + L) Alexa Fluor^®^ 647 (A21445, Life Technologies, Carlsbad, CA, USA), diluted 1:15,000. The readout was performed by a laser scanner (InnoScan^®^ 1100, Innopsys, Chicago, IL, USA). Image analysis and quality control of spots were performed by GenePix Pro 5.1 (Molecular Devices LLC, San Jose, CA, USA).

### 4.4. Targeted Autoantibody Screening Using Antigen Bead Array

A targeted antigen bead array was then generated with protein fragments representing the selected proteins and used to test 55 SSc plasma samples and 52 controls as previously described [14].

In detail, 246 antigens, of which 73 were selected based on the planar microarray analysis and 173 representing proteins of interest in the context of SSc, were immobilized on the surface of uniquely color-coded magnetic beads (MagPlex, Luminex Corp., Austin, TX, USA). Beads were then mixed to generate a bead array that was applied to test plasma samples from 55 SSc cases and 52 controls, as previously described [14]. Briefly, samples were diluted 1:250 in assay buffer (PBS-T 0.05%, 3% (*w*/*v*) BSA, 5% (*w*/*v*) skim-milk powder, supplemented with 160 µg/mL His6ABP), pre-incubated at room temperature for 1 h to block potential tag-reactive antibodies, and applied on the bead-array at room temperature for 2 h. The excess sample was then washed away using PBS-T 0.05%. Next, a cross-linking step with 0.2% paraformaldehyde was performed to stabilize the antigen-autoantibodies immunocomplexes, followed by incubation with 0.4 µg/mL R-PE conjugated anti-human IgG detection antibody (󠄁H10104, Invitrogen, Waltham, MA, USA) for 30 min at room temperature. The readout was performed using a FLEXMAP 3D^®^ instrument (Luminex Corp., Austin, TX, USA).

### 4.5. Antigens

The antigens included in this study are protein fragments (80–100 amino acids) produced in *E. coli* as a fusion to His6-ABP and MS-verified within the Human Protein Atlas project (www.proteinatlas.org, accessed on 14 March 2023).

### 4.6. Data Analysis

R studio version 4.0.4 was used for the data analysis. The data generated from the planar antigen array were analyzed with a standardized workflow developed in-house [14]. The raw data were normalized and transformed in nSD (number of standard deviations from the array specific mean intensity) using the formula: nSD = (xi − mean(xn))/SD(xn), where xi is the raw intensity signal of the single protein fragment on the array, mean(xn) is the mean of the raw intensity signals across the array, and SD(xn) is the standard deviation across the array. A 4SD cutoff was chosen for IgG binding signal intensity. Autoantibodies passing the cutoff were ranked based on their intensity signals and the width of the intensity signal difference between the two pools. Considering our interest in identifying autoantibodies targeting fibrosis, we selected the autoantibodies showing higher intensity signals in samples of patients with dcSSc to be passed on to verification by bead array.

The bead-array intensity data were normalized to correct for sample-specific background levels as previously published [14]. The applied formula is: nMAD = (xi − median(xn))/MAD(xn), where nMAD is the number of median absolute deviations (MAD) from the sample median, xi is the raw intensity signal of the single protein fragment in the sample, median(xn) is the median intensity signal across the sample, and MAD(xn) is the MAD across the sample. A cutoff was set based on the distribution of the intensity signals for each antigen across all samples. Samples passing the cutoff were defined as reactive (assigned a value of 1) for that specific autoantibody, while samples not passing the cutoff were considered as negative (assigned a value of 0) for that specific autoantibody. Fisher´s Exact test was applied to the binary data to compare the prevalence of specific autoantibodies in SSc patients and in controls as well as between subgroups of patients. Heatmaps and cluster analysis was applied to combine autoantibodies and possibly identify signatures associated with specific clinical features. The ROC curve was used to test the performance of autoantibody combinations in classifying SSc patients and controls in the tested cohort. Mann–Whitney–Wilcoxon test was used to compare the autoantibody load (number of autoantibodies per sample) between cases and controls, as well as between patients with and without fibrosis. Kruskal–Wallis test was applied to compare the autoantibody load when more than two groups are considered.

The generated data were analyzed focusing on three main aims. Initially, we evaluated the autoantibody load in patients and controls to determine whether we could see any general higher reactivity in SSc or in specific patient subgroups. Second, we focused on single autoantibodies to identify whether any showed higher prevalence in SSc. Finally, we aimed to link autoantibodies, as single or in combination, to clinical features to test their potential to subclassify the SSc cohort.

## Figures and Tables

**Table 1:** Study group: Clinical characteristics and clinical autoantibody status.

Study Group	SSc Patients	Controls
Number, N	55	52
Female/male, N (%)	46 (84%)/9 (16%)	39 (75%)/13 (25%)
Age, median (range)	61 (25–85)	53 (21–79)
Limited SSc/diffuse SSc/no sclerosis, N	42/11/2	-
Disease duration, Y, median (range)	7 (0–37)	
mRSScore, median (range)	9 (0–46)	-
Autoantibodies, N		
Anti-Scl70	18	-
Anti-centromere	19	-
Anti-SSA/Ro	4	-
Anti-Rnp/Sm	3	-
Negative to anti-Scl70 and -centromere	14	-
Clinical manifestations, N		
Calcinosis cutis	16	-
Digital ulcers	12	-
Dysphagia	29	-
Lung fibrosis	14	-
PAH	20	-
Raynaud	47	-
Reflux	30	-
Sicca	16	-

## Data Availability

Data cannot be shared publicly as they contain sensitive personal information, which is protected by the GDPR. Data are available on request from the SciLifeLab Data Repository (10.17044/scilifelab.22269211) for researchers who meet the criteria for access to sensitive personal data. Code cannot be shared publicly as sensitive personal information, which is protected by the GDPR, can be inferred. Code is available on request from the SciLifeLab Data Repository (10.17044/scilifelab.22269211) for researchers who meet the criteria for access to sensitive personal data.

## Supplementary Materials

The following supporting information can be downloaded at: https://www.mdpi.com/article/10.3390/ijms24065629/s1.
