# Supplementary material for: Autoantibodies against PIP4K2B and AKT3 Are Associated with Skin and Lung Fibrosis in Patients with Systemic Sclerosis
Source: Int J Mol Sci. 2023 Mar 15;24(6):5629. doi: 10.3390/ijms24065629 (PMC10051301; doi:10.3390/ijms24065629)
Supplement: Supplementary file 1 [file ijms-24-05629-s001.zip › ijms-2262389-supplementary.pdf]

Supplementary Material

SUPPLEMENTARY FIGURES AND LEGENDS

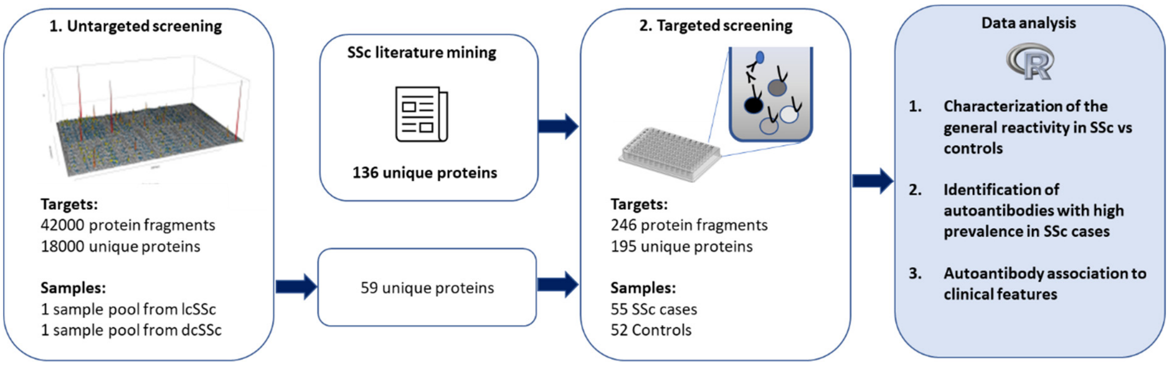

Supplementary Figure S1. Study design. In the first phase, we screened two plasma pools each including samples from 4 patients with localized SSc (lcSSc) or diffuse SSc (dcSSc) using a planar antigen array to identify IgG binding to protein fragments representing 18,000 human proteins. Next, a targeted screening was designed where a bead array was generated with protein fragments representing the protein selection from the planar array analysis plus a selection from literature and tested on a cohort of plasma samples including 55 SSc patients and 52 controls. The generated data were analyzed using the R studio statistical environment. The data analysis focused on identifying autoantibodies highly prevalent in SSc that could be associated with clinical features, especially fibrosis.

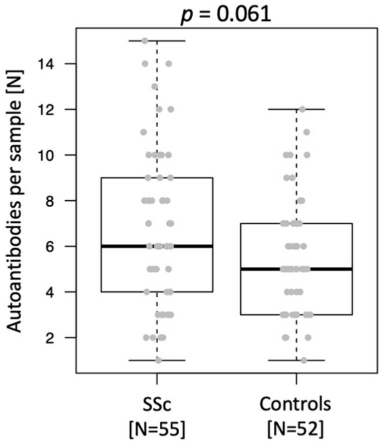

Supplementary Figure S2. Autoantibody load in plasma of SSc patients and controls. The plasma IgG reactivity towards 246 protein fragments was determined using an antigen bead array. The data showed a higher, even though not significant (Fisher´s exact test), autoantibody load in SSc patients compared to controls.

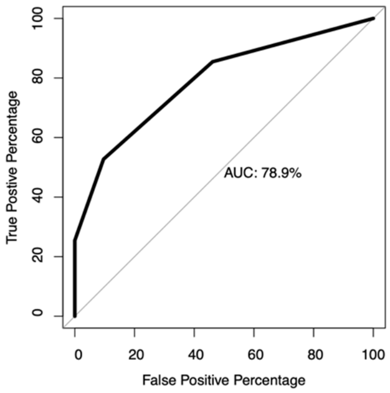

Supplementary Figure S3. ROC curve analysis of the 11-autoantibody panel. The curve shows the performance of the 11-autoantibody panel in separating SSc cases from controls in the tested cohort. The curve represents the True Positive and False Positive rates resulting from setting cutoffs of ≥1-3 autoantibodies per sample to classify SSc patients and controls.

SUPPLEMENTARY TABLES

Supplementary Table S1. Sample pools from patients with dcSSc and lcSSc: clinical characteristics and clinical autoantibody status.

Pools	dcSSc	lcSSc
Number, N	4	4
Female/ male, N (%)	2 (50%)/ 2 (50%)	2 (50%)/ 2 (50%)
Age, median (range)	50 (41-65)	57 (41-65)
Disease duration, Y, median (range)	2.5 (1-15)	5.5 (2-16)
mRSScore, median (range)	32 (17-46)	5 (2-12)
Autoantibodies, N		
Anti-Scl70	3	1
Anti-centromere	1	3
Anti-SSA/Ro	1	0
Anti-Rnp/Sm	0	0
Negative to anti-Scl70 and -centromere	1	0
Clinical manifestations, N		
Calcinosis cutis	0	2
Digital ulcers	1	2
Dysphagia	2	2
Lung fibrosis	2	0
PAH	2	0
Raynaud	3	4
Reflux	2	3
Sicca	0	1

Supplementary Table S2. Antigens included in the targeted screening.

	Antigen code	Gene	Uniprot ID	Gene description	Selection	Ensembl Gene ID
1	HPRR3140513	A1BG	P04217	alpha-1-B glycoprotein	Untargeted screening	ENSG00000121410

2	HPRR3140512	A1BG	P04217	alpha-1-B glycoprotein	Untargeted screening	ENSG00000121410
3	HPRR2750140	ABL1	P00519	ABL proto-oncogene 1, non-receptor tyrosine kinase	Literature	ENSG00000097007
4	HPRR3051133	ACCSL	Q4AC99	1-aminocyclopropane-1-carboxylate synthase homolog (inactive) like	Untargeted screening	ENSG00000205126
5	HPRR2551494	ACP6	Q9NPH0	acid phosphatase 6, lysophosphatidic	Literature	ENSG00000162836
6	HPRR2640138	ADAM12	O43184	ADAM metallopeptidase domain 12	Literature	ENSG00000148848
7	HPRR2640136	ADAM12	O43184	ADAM metallopeptidase domain 12	Literature	ENSG00000148848
8	HPRR3790319	ADSS1/ADSSL1	Q8N142	adenylosuccinate synthase 1	Untargeted screening	ENSG00000185100
9	HPRR370232	AGTR1	P30556	angiotensin II receptor type 1	Literature	ENSG00000144891
10	HPRR141612	AKT1	P31749	AKT serine/threonine kinase 1	Literature	ENSG00000142208
11	HPRR3520031	AKT2	P31751	AKT serine/threonine kinase 2	Literature	ENSG00000105221
12	HPRR2690020	AKT3	Q9Y243	AKT serine/threonine kinase 3	Literature	ENSG00000117020

13	HPRR3070036	ANO2	Q9NQ90	anoctamin 2	Untargete d screening	ENSG0000004761 7
14	HPRR3070037	ANO2	Q9NQ90	anoctamin 2	Untargete d screening	ENSG0000004761 7
15	HPRR3300295	ASIP	P42127	agouti signaling protein	Literature	ENSG0000010144 0
16	HPRR4000319	AXIN2	Q9Y2T1	axin 2	Literature	ENSG0000016864 6
17	HPRR2470463	CACNA1B	Q00975	calcium voltage-gated channel subunit alpha1 B	Untargete d screening	ENSG0000014840 8
18	HPRR3410011	CACNA1S	Q13698	calcium voltage-gated channel subunit alpha1 S	Untargete d screening	ENSG0000008124 8
19	HPRR3790673	CASTOR2/GASTL 2	A6NHX0	cytosolic arginine sensor for mTORC1 subunit 2	Untargete d screening	ENSG0000027407 0
20	HPRR3790301	CCDC137	Q6PK04	coiled-coil domain containing 137	Untargete d screening	ENSG0000018529 8
21	HPRR3690018	CCL18	P55774	C-C motif chemokine ligand 18	Literature	ENSG0000027538 5
22	HPRR3500294	CCL7	P80098	C-C motif chemokine ligand 7	Literature	ENSG0000010868 8
23	HPRR3420436	CD163	Q86VB7	CD163 molecule	Literature	ENSG0000017757 5
24	HPRR2990191	CD34	P28906	CD34 molecule	Literature	ENSG0000017405 9
25	HPRR2990193	CD34	P28906	CD34 molecule	Literature	ENSG0000017405 9

26	HPRR2990083	CD80	P33681	CD80 molecule	Literature	ENSG00000121594
27	HPRR1450199	CD86	P42081	CD86 molecule	Literature	ENSG00000114013
28	HPRR4240093	CDH5	P33151	cadherin 5	Literature	ENSG00000179776
29	HPRR3890121	CENPA	P49450	centromere protein A	Literature	ENSG00000115163
30	HPRR3310037	CENPB	P07199	centromere protein B	Literature	ENSG00000125817
31	HPRR2090090	CHRM3	P20309	cholinergic receptor muscarinic 3	Literature	ENSG00000133019
32	HPRR1160007	CNR1	P21554	cannabinoid receptor 1	Literature	ENSG00000118432
33	HPRR1160013	CNR2	P34972	cannabinoid receptor 2	Literature	ENSG00000188822
34	HPRR2890225	COL18A1	P39060	collagen type XVIII alpha 1 chain	Literature	ENSG00000182871
35	HPRR2890226	COL18A1	P39060	collagen type XVIII alpha 1 chain	Literature	ENSG00000182871
36	HPRR1770038	COL1A1	P02452	collagen type I alpha 1 chain	Literature	ENSG00000108821
37	HPRR4030210	COL1A2	P08123	collagen type I alpha 2 chain	Literature	ENSG00000164692
38	HPRR4030209	COL1A2	P08123	collagen type I alpha 2 chain	Literature	ENSG00000164692
39	HPRR1770057	COL5A1	P20908	collagen type V alpha 1 chain	Literature	ENSG00000130635
40	HPRR3250149	COMP	P49747	cartilage oligomeric matrix protein	Literature	ENSG00000105664

41	HPRR3860038	CREBBP	Q92793	CREB binding protein	Literature	ENSG00000005339
42	HPRR3970260	CSF2	P04141	colony stimulating factor 2	Literature	ENSG00000164400
43	HPRR2760285	CSF2	P04141	colony stimulating factor 2	Literature	ENSG00000164400
44	HPRR2590050	CCN2/CTGF	P29279	Cellular Communication Network Factor 2 or Connective Tissue Growth Factor	Literature	ENSG00000118523
45	HPRR2590049	CCN2/CTGF	P29279	Cellular Communication Network Factor 2 or Connective Tissue Growth Factor	Literature	ENSG00000118523
46	HPRR2760300	CTNNB1	P35222	catenin beta 1	Literature	ENSG00000168036
47	HPRR2700195	CXCL8	P10145	C-X-C motif chemokine ligand 8	Literature	ENSG00000169429
48	HPRR3720504	DIDO1	Q9BTC0	death inducer-obliterator 1	Untargeted screening	ENSG00000101191
49	HPRR3880140	DKK1	O94907	dickkopf WNT signaling pathway inhibitor 1	Literature	ENSG00000107984
50	HPRR2570091	EDN1	P05305	endothelin 1	Literature	ENSG00000078401
51	HPRR2570089	EDN1	P05305	endothelin 1	Literature	ENSG00000078401
52	HPRR2090126	EDNRA	P25101	endothelin receptor type A	Literature	ENSG00000151617
53	HPRR2090127	EDNRA	P25101	endothelin receptor type A	Literature	ENSG00000151617

54	HPRR2760156	EGR1	P18146	early growth response 1	Literature	ENSG0000012073 8
55	HPRR4120223	EID2	Q8N6I1	EP300 interacting inhibitor of differentiation 2	Untargete d screening	ENSG0000017639 6
56	HPRR3280312	EID2	Q8N6I1	EP300 interacting inhibitor of differentiation 2	Untargete d screening	ENSG0000017639 6
57	HPRR1950396	ENG	P17813	endoglin	Literature	ENSG0000010699 1
58	HPRR4170053	ENG	P17813	endoglin	Literature	ENSG0000010699 1
59	HPRR221232	EP300	Q09472	E1A binding protein p300	Literature	ENSG0000010039 3
60	HPRR4180964	ETS1	P14921	ETS proto-oncogene 1, transcription factor	Literature	ENSG0000013495 4
61	HPRR2050366	FBN1	P35555	fibrillin 1	Literature	ENSG0000016614 7
62	HPRR2050365	FBN1	P35555	fibrillin 1	Literature	ENSG0000016614 7
63	HPRR3060143	FBXW8	Q8N3Y1	F-box and WD repeat domain containing 8	Untargete d screening	ENSG0000017498 9
64	HPRR3830253	FGF2	P09038	fibroblast growth factor 2	Literature	ENSG0000013868 5
65	HPRR4290340	FGF2	P09038	fibroblast growth factor 2	Literature	ENSG0000013868 5
66	HPRR3870004	FGFR1	P11362	fibroblast growth factor receptor 1	Literature	ENSG0000007778 2
67	HPRR3760448	FLI1	Q01543	Fli-1 proto-oncogene, ETS transcription factor	Literature	ENSG0000015170 2

68	HPRR2050095	FN1	P02751	fibronectin 1	Literature	ENSG0000011541 4
69	HPRR2090190	FSHR	P23945	follicle stimulating hormone receptor	Untargete d screening	ENSG0000017082 0
70	HPRR4220457	FSHR	P23945	follicle stimulating hormone receptor	Untargete d screening	ENSG0000017082 0
71	HPRR1950254	FXYD5	Q96DB9	FXYD domain containing ion transport regulator 5	Untargete d screening	ENSG0000008932 7
72	HPRR2810079	GABRE	P78334	gamma-aminobutyric acid type A receptor subunit epsilon	Untargete d screening	ENSG0000010228 7
73	HPRR3140486	GLE1	Q53GS7	GLE1 RNA export mediator	Untargete d screening	ENSG0000011939 2
74	HPRR3470107	GRXCR2	A6NFK2	glutaredoxin and cysteine rich domain containing 2	Untargete d screening	ENSG0000020492 8
75	HPRR3300109	GSS	P48637	glutathione synthetase	Untargete d screening	ENSG0000010098 3
76	HPRR2780009	HDAC1	Q13547	histone deacetylase 1	Literature	ENSG0000011647 8
77	HPRR2370166	HELZ	P42694	helicase with zinc finger	Untargete d screening	ENSG0000019826 5
78	HPRR3140054	HSPA5	P11021	heat shock protein family A (Hsp70) member 5	Untargete d screening	ENSG0000004457 4
79	HPRR1220005	HTR5A	P47898	5-hydroxytryptamine receptor 5A	Literature	ENSG0000015721 9

80	HPRR4160047	ICA1	Q05084	islet cell autoantigen 1	Untargeted screening	ENSG00000003147
81	HPRR1510003	ICA1	Q05084	islet cell autoantigen 1	Untargeted screening	ENSG00000003147
82	HPRR330202	ICAM1	P05362	intercellular adhesion molecule 1	Literature	ENSG00000090339
83	HPRR330201	ICAM1	P05362	intercellular adhesion molecule 1	Literature	ENSG00000090339
84	HPRR370176	IFI16	Q16666	interferon gamma inducible protein 16	Literature	ENSG00000163565
85	HPRR4190871	IFI16	Q16666	interferon gamma inducible protein 16	Literature	ENSG00000163565
86	HPRR2760324	IFNB1	P01574	interferon beta 1	Literature	ENSG00000171855
87	HPRR2960107	IGF2BP2	Q9Y6M1	insulin like growth factor 2 mRNA binding protein 2	Untargeted screening	ENSG00000073792
88	HPRR2760042	IL11	P20809	interleukin 11	Literature	ENSG00000095752
89	HPRR330015	IL13	P35225	interleukin 13	Literature	ENSG00000169194
90	HPRR2760112	IL17A	Q16552	interleukin 17A	Literature	ENSG00000112115
91	HPRR4180350	IL1A	P01583	interleukin 1 alpha	Literature	ENSG00000115008
92	HPRR330003	IL1B	P01584	interleukin 1 beta	Literature	ENSG00000125538
93	HPRR4370105	IL1B	P01584	interleukin 1 beta	Literature	ENSG00000125538

94	HPRR3071105	IL31	Q6EBC2	interleukin 31	Literature	ENSG0000020467 1
95	HPRR3920122	IL4	P05112	interleukin 4	Literature	ENSG0000011352 0
96	HPRR330007	IL6	P05231	interleukin 6	Literature	ENSG0000013624 4
97	HPRR2760215	IL6	P05231	interleukin 6	Literature	ENSG0000013624 4
98	HPRR4180952	IL6ST	P40189	interleukin 6 signal transducer	Literature	ENSG0000013435 2
99	HPRR550014	ILK	Q13418	integrin linked kinase	Literature	ENSG0000016633 3
100	HPRR3130024	ILK	Q13418	integrin linked kinase	Literature	ENSG0000016633 3
101	HPRR610018	IRF3	Q14653	interferon regulatory factor 3	Literature	ENSG0000012645 6
102	HPRR4340115	IRF5	Q13568	interferon regulatory factor 5	Literature	ENSG0000012860 4
103	HPRR2700232	ITGA1	P56199	integrin subunit alpha 1	Literature	ENSG0000021394 9
104	HPRR550012	ITGA5	P08648	integrin subunit alpha 5	Literature	ENSG0000016163 8
105	HPRR3760412	ITGB1	P05556	integrin subunit beta 1	Literature	ENSG0000015009 3
106	HPRR3760413	ITGB1	P05556	integrin subunit beta 1	Literature	ENSG0000015009 3
107	HPRR1310041	ITGB6	P18564	integrin subunit beta 6	Literature	ENSG0000011522 1
108	HPRR2970027	JAK2	O60674	Janus kinase 2	Literature	ENSG0000009696 8

109	HPRR2970026	JAK2	O60674	Janus kinase 2	Literature	ENSG0000009696 8
110	HPRR3460601	JAKMIP2	Q96AA8	janus kinase and microtubule interacting protein 2	Untargete d screening	ENSG0000017604 9
111	HPRR4030465	KCNB2	Q92953	potassium voltage-gated channel subfamily B member 2	Untargete d screening	ENSG0000018267 4
112	HPRR2960533	KIF15	Q9NS87	kinesin family member 15	Untargete d screening	ENSG0000016380 8
113	HPRR2960534	KIF15	Q9NS87	kinesin family member 15	Untargete d screening	ENSG0000016380 8
114	HPRR2740008	KIF4A/B	O95239	kinesin family member 4A/B	Literature	ENSG0000009088 9
115	HPRR1950865	KL	Q9UEF7	klotho	Untargete d screening	ENSG0000013311 6
116	HPRR4180849	KL	Q9UEF7	klotho	Untargete d screening	ENSG0000013311 6
117	HPRR680113	KLF1	Q13351	Kruppel like factor 1	Untargete d screening	ENSG0000010561 0
118	HPRR3720637	KLF1	Q13351	Kruppel like factor 1	Untargete d screening	ENSG0000010561 0
119	HPRR3920097	KLF2	Q9Y5W3	Kruppel like factor 2	Untargete d screening	ENSG0000012752 8
120	HPRR3920099	KLF2	Q9Y5W3	Kruppel like factor 2	Untargete d screening	ENSG0000012752 8

121	HPRR4180438	KMT2A	Q03164	lysine methyltransferase 2A	Untargeted screening	ENSG00000118058
122	HPRR2501239	KRBA2	Q6ZNG9	KRAB-A domain containing 2	Untargeted screening	ENSG00000184619
123	HPRR2980021	LATS1	O95835	large tumor suppressor kinase 1	Untargeted screening	ENSG00000131023
124	HPRR360140	LEF1	Q9UJU2	lymphoid enhancer binding factor 1	Literature	ENSG00000138795
125	HPRR3010255	LOX	P28300	lysyl oxidase	Literature	ENSG00000113083
126	HPRR3010254	LOX	P28300	lysyl oxidase	Literature	ENSG00000113083
127	HPRR1410088	LPAR1	Q92633	lysophosphatidic acid receptor 1	Literature	ENSG00000198121
128	HPRR4280266	LPAR1	Q92633	lysophosphatidic acid receptor 1	Literature	ENSG00000198121
129	HPRR650100	LRP1	Q07954	LDL receptor related protein 1	Literature	ENSG00000123384
130	HPRR4290189	LYPLA1	O75608	lysophospholipase 1	Untargeted screening	ENSG00000120992
131	HPRR2690048	MAP2K1	Q02750	mitogen-activated protein kinase kinase 1	Literature	ENSG00000169032
132	HPRR3770077	MAPK14	Q16539	mitogen-activated protein kinase 14	Literature	ENSG00000112062
133	HPRR3880317	MAPK14	Q16539	mitogen-activated protein kinase 14	Literature	ENSG00000112062
134	HPRR2760026	MCM2	P49736	minichromosome maintenance	Untargeted screening	ENSG00000073111

				complex component 2		
135	HPRR3830164	MED1	Q15648	mediator complex subunit 1	Untargete d screening	ENSG0000012568 6
136	HPRR3120181	MEX3C	Q5U5Q3	mex-3 RNA binding family member C	Untargete d screening	ENSG0000017662 4
137	HPRR221459	MRTFA/MKL1	Q969V6	myocardin related transcription factor A	Literature	ENSG0000019658 8
138	HPRR1450602	MRTFB/MKL2	Q9ULH7	myocardin related transcription factor B	Literature	ENSG0000018626 0
139	HPRR1320028	MMP1	P03956	matrix metallopeptidase 1	Literature	ENSG0000019661 1
140	HPRR3770068	MMP1	P03956	matrix metallopeptidase 1	Literature	ENSG0000019661 1
141	HPRR3140420	MMP12	P39900	matrix metallopeptidase 12	Literature	ENSG0000026240 6
142	HPRR3140418	MMP12	P39900	matrix metallopeptidase 12	Literature	ENSG0000026240 6
143	HPRR1320019	MMP3	P08254	matrix metallopeptidase 3	Literature	ENSG0000014996 8
144	HPRR3440044	MMP3	P08254	matrix metallopeptidase 3	Literature	ENSG0000014996 8
145	HPRR3760072	MMP7	P09237	matrix metallopeptidase 7	Literature	ENSG0000013767 3
146	HPRR4110413	MMP7	P09237	matrix metallopeptidase 7	Literature	ENSG0000013767 3
147	HPRR330062	MMP9	P14780	matrix metallopeptidase 9	Literature	ENSG0000010098 5

148	HPRR4160805	MMP9	P14780	matrix metallopeptidase 9	Literature	ENSG00000100985
149	HPRR2150148	MS4A1	P11836	membrane spanning 4-domains A1	Literature	ENSG00000156738
150	HPRR1420191	MUC1	P15941	mucin 1, cell surface associated	Literature	ENSG00000185499
151	HPRR2460052	MYD88	Q99836	MYD88 innate immune signal transduction adaptor	Literature	ENSG00000172936
152	HPRR4030408	NDUFA11	Q86Y39	NADH:ubiquinone oxidoreductase subunit A11	Literature	ENSG00000174886
153	HPRR4220008	NFAT5	O94916	nuclear factor of activated T cells 5	Untargeted screening	ENSG00000102908
154	HPRR3070953	NOC4L	Q9BVI4	nucleolar complex associated 4 homolog	Untargeted screening	ENSG00000184967
155	HPRR2440552	NOM1	Q5C9Z4	nucleolar protein with MIF4G domain 1	Untargeted screening	ENSG00000146909
156	HPRR4290379	NOTCH1	P46531	notch receptor 1	Literature	ENSG00000148400
157	HPRR260118	NOTCH2	Q04721	notch receptor 2	Literature	ENSG00000134250
158	HPRR260117	NOTCH2	Q04721	notch receptor 2	Literature	ENSG00000134250
159	HPRR2170028	NOX4	Q9NPH5	NADPH oxidase 4	Literature	ENSG00000086991
160	HPRR2170027	NOX4	Q9NPH5	NADPH oxidase 4	Literature	ENSG00000086991

161	HPRR3730343	NR4A1	P22736	nuclear receptor subfamily 4 group A member 1	Literature	ENSG00000123358
162	HPRR3730197	NT5C1A	Q9BXI3	5'-nucleotidase, cytosolic IA	Literature	ENSG00000116981
163	HPRR3730198	NT5C1A	Q9BXI3	5'-nucleotidase, cytosolic IA	Literature	ENSG00000116981
164	HPRR221161	OSM	P13725	oncostatin M	Literature	ENSG00000099985
165	HPRR3190230	PAGR1	Q9BTK6	PAXIP1 associated glutamate rich protein 1	Untargeted screening	ENSG00000280789
166	HPRR670206	PDE5A	O76074	phosphodiesterase 5A	Literature	ENSG00000138735
167	HPRR670205	PDE5A	O76074	phosphodiesterase 5A	Literature	ENSG00000138735
168	HPRR3160287	PDF	Q9HBH1	peptide deformylase, mitochondrial	Untargeted screening	ENSG00000258429
169	HPRR670190	PDGFRA	P16234	platelet derived growth factor receptor alpha	Literature	ENSG00000134853
170	HPRR2750035	PDGFRB	P09619	platelet derived growth factor receptor beta	Literature	ENSG00000113721
171	HPRR3030043	PDPK1	O15530	3-phosphoinositide dependent protein kinase 1	Literature	ENSG00000140992
172	HPRR1470131	PHC1	P78364	polyhomeotic homolog 1	Untargeted screening	ENSG00000111752
173	HPRR1810044	PHYHD1	Q5SRE7	phytanoyl-CoA dioxygenase	Untargeted screening	ENSG00000175287

				domain containing 1		
174	HPRR4190558	PIK3R1	P27986	phosphoinositide-3-kinase regulatory subunit 1	Literature	ENSG00000145675
175	HPRR3930144	PIP4K2B	P78356	phosphatidylinositol-5-phosphate 4-kinase type 2 beta	Untargeted screening	ENSG00000276293
176	HPRR3020346	POLR3A	O14802	RNA polymerase III subunit A	Literature	ENSG00000148606
177	HPRR1450429	POLR3K	Q9Y2Y1	RNA polymerase III subunit K	Literature	ENSG00000161980
178	HPRR3700267	PRKCG	P05129	protein kinase C gamma	Literature	ENSG00000126583
179	HPRR2770085	PTGER1	P34995	prostaglandin E receptor 1	Literature	ENSG00000160951
180	HPRR2710019	PTGIR	P43119	prostaglandin I2 receptor	Literature	ENSG00000160013
181	HPRR490039	PTK2	Q05397	protein tyrosine kinase 2	Literature	ENSG00000169398
182	HPRR3770043	PTPRS	Q13332	protein tyrosine phosphatase receptor type S	Untargeted screening	ENSG00000105426
183	HPRR2360025	RASAL2	Q9UJF2	RAS protein activator like 2	Untargeted screening	ENSG00000075391
184	HPRR2360023	RASAL2	Q9UJF2	RAS protein activator like 2	Untargeted screening	ENSG00000075391
185	HPRR4050163	RNPC3	Q96LT9	RNA binding region (RNP1, RRM) containing 3	Literature	ENSG00000185946
186	HPRR490004	ROCK1	Q13464	Rho associated coiled-coil	Literature	ENSG00000067900

				containing protein kinase 1		
187	HPRR3390156	ROCK1	Q13464	Rho associated coiled-coil containing protein kinase 1	Literature	ENSG00000067900
188	HPRR1840080	ROCK2	O75116	Rho associated coiled-coil containing protein kinase 2	Literature	ENSG00000134318
189	HPRR1840079	ROCK2	O75116	Rho associated coiled-coil containing protein kinase 2	Literature	ENSG00000134318
190	HPRR2850065	SAMD15	Q9P1V8	sterile alpha motif domain containing 15	Untargeted screening	ENSG00000100583
191	HPRR2551271	SASS6	Q6UVJ0	SAS-6 centriolar assembly protein	Untargeted screening	ENSG00000156876
192	HPRR3250185	SCAF1	Q9H7N4	SR-related CTD associated factor 1	Untargeted screening	ENSG00000126461
193	HPRR2550019	SELE	P16581	selectin E	Literature	ENSG00000007908
194	HPRR2550021	SELE	P16581	selectin E	Literature	ENSG00000007908
195	HPRR3420665	SELL	P14151	selectin L	Untargeted screening	ENSG00000188404
196	HPRR3420664	SELL	P14151	selectin L	Untargeted screening	ENSG00000188404
197	HPRR3790545	SERPINB13	Q9UIV8	serpin family B member 13	Untargeted screening	ENSG00000197641

198	HPRR3790544	SERPINB13	Q9UIV8	serpin family B member 13	Untargeted screening	ENSG00000197641
199	HPRR3020262	SFTPD	P35247	surfactant protein D	Literature	ENSG00000133661
200	HPRR3890703	SFTPD	P35247	surfactant protein D	Literature	ENSG00000133661
201	HPRR3140530	SGPP1	Q9BX95	sphingosine-1-phosphate phosphatase 1	Literature	ENSG00000126821
202	HPRR3141067	SHH	Q15465	sonic hedgehog signaling molecule	Literature	ENSG00000164690
203	HPRR3870113	SLC17A6	Q9P2U8	solute carrier family 17 member 6	Untargeted screening	ENSG00000091664
204	HPRR3760231	SLC24A4	Q8NFF2	solute carrier family 24 member 4	Untargeted screening	ENSG00000140090
205	HPRR3430060	SMAD1	Q15797	SMAD family member 1	Literature	ENSG00000170365
206	HPRR4080072	SMAD2	Q15796	SMAD family member 2	Literature	ENSG00000175387
207	HPRR3340151	SMAD3	P84022	SMAD family member 3	Literature	ENSG00000166949
208	HPRR630026	SMAD4	Q13485	SMAD family member 4	Literature	ENSG00000141646
209	HPRR640097	SMAD7	O15105	SMAD family member 7	Literature	ENSG00000101665
210	HPRR3210218	SNRNP70	P08621	small nuclear ribonucleoprotein U1 subunit 70	Literature	ENSG00000104852
211	HPRR3310244	SPTLC3	Q9NUV7	serine palmitoyltransferase	Untargeted screening	ENSG00000172296

				long chain base subunit 3		
212	HPRR2970130	SRC	P12931	SRC proto-oncogene, non-receptor tyrosine kinase	Literature	ENSG00000197122
213	HPRR260287	STAT4	Q14765	signal transducer and activator of transcription 4	Literature	ENSG00000138378
214	HPRR1450666	SUPT3H	O75486	SPT3 homolog, SAGA and STAGA complex component	Untargeted screening	ENSG00000196284
215	HPRR2800078	TAB1	Q15750	TGF-beta activated kinase 1 (MAP3K7) binding protein 1	Literature	ENSG00000100324
216	HPRR4080105	TEAD1	P28347	TEA domain transcription factor 1	Literature	ENSG00000187079
217	HPRR3700118	TGFB1		transforming growth factor beta 1	Literature	ENSG00000105329
218	HPRR2110073	TGFBR1	P36897	transforming growth factor beta receptor 1	Literature	ENSG00000106799
219	HPRR2430030	TGM2	P21980	transglutaminase 2	Literature	ENSG00000198959
220	HPRR350066	THBS1	P07996	thrombospondin 1	Literature	ENSG00000137801
221	HPRR4190052	THBS1	P07996	thrombospondin 1	Literature	ENSG00000137801
222	HPRR3250232	TICAM1	Q8IUC6	toll like receptor adaptor molecule 1	Literature	ENSG00000127666
223	HPRR3700361	TLR4	O00206	toll like receptor 4	Literature	ENSG00000136869

224	HPRR670038	TNC	P24821	tenascin C	Literature	ENSG0000004198 2
225	HPRR2420092	TOP1	P11387	DNA topoisomerase I	Literature	ENSG0000019890 0
226	HPRR3340413	TRIM10	Q9UDY6	tripartite motif containing 10	Untargete d screening	ENSG0000020461 3
227	HPRR3340414	TRIM10	Q9UDY6	tripartite motif containing 10	Untargete d screening	ENSG0000020461 3
228	HPRR1060031	TRIM21	P19474	tripartite motif containing 21	Literature	ENSG0000013210 9
229	HPRR400060	RO60/TROVE2	P10155	Ro60, Y RNA binding protein	Literature	ENSG0000011674 7
230	HPRR1440120	USP30	Q70CQ3	ubiquitin specific peptidase 30	Untargete d screening	ENSG0000013509 3
231	HPRR2760266	VCAM1	P19320	vascular cell adhesion molecule 1	Literature	ENSG0000016269 2
232	HPRR2760265	VCAM1	P19320	vascular cell adhesion molecule 1	Literature	ENSG0000016269 2
233	HPRR3730128	VEGFA	P15692	vascular endothelial growth factor A	Literature	ENSG0000011271 5
234	HPRR4040205	VEGFB	P49765	vascular endothelial growth factor B	Literature	ENSG0000017351 1
235	HPRR3760438	VEGFC	P49767	vascular endothelial growth factor C	Literature	ENSG0000015063 0
236	HPRR230623	VEGFD	O43915	vascular endothelial growth factor D	Literature	ENSG0000016519 7
237	HPRR4160898	VSX1	Q9NZR4	visual system homeobox 1	Untargete d screening	ENSG0000010098 7

238	HPRR330185	VWF	P04275	von Willebrand factor	Literature	ENSG0000011079 9
239	HPRR330186	VWF	P04275	von Willebrand factor	Literature	ENSG0000011079 9
240	HPRR370161	WIF1	Q9Y5W5	WNT inhibitory factor 1	Literature	ENSG0000015607 6
241	HPRR2300013	CCN6/WISP3	O95389	cellular communication network factor 6 OR WNT1 Inducible Signaling Pathway Protein 3	Untargete d screening	ENSG0000011276 1
242	HPRR2300016	CCN6/WISP3	O95389	cellular communication network factor 6 OR WNT1 Inducible Signaling Pathway Protein 3	Untargete d screening	ENSG0000011276 1
243	HPRR2510003	WIZ	O95785	WIZ zinc finger	Untargete d screening	ENSG0000001145 1
244	HPRR1450035	WWTR1	Q9GZV5	WW domain containing transcription regulator 1	Literature	ENSG0000001840 8
245	HPRR3050310	YAP1	P46937	Yes1 associated transcriptional regulator	Literature	ENSG0000013769 3
246	HPRR4030212	ZNF202	O95125	zinc finger protein 202	Untargete d screening	ENSG0000016626 1

Supplementary Table S3. Performance of the 11 autoantibodies in classifying SSc patients and controls at different cutoffs.

	SSc patients (N=55)			Controls (N=52)		
Cutoff [N of detected autoantibodies]	Positive results [N]	Negative results [N]	True positive results [%]	Positive results [N]	Negative results [N]	False positive results [%]
≥3	14	41	25	0	52	0
≥2	29	26	53	5	47	10
≥1	47	8	85	24	28	46
